# Full-Genome Sequencing of a Virus from a 33-Year-Old Sample Demonstrates that *Arracacha Mottle Virus* Is Synonymous with *Arracacha Virus Y*

**DOI:** 10.1128/MRA.01393-18

**Published:** 2018-11-29

**Authors:** Ian P. Adams, Neil Boonham, Roger A. C. Jones

**Affiliations:** a Fera Science Ltd., Sand Hutton, York, United Kingdom; b School of Natural and Environmental Sciences, Newcastle University, Newcastle upon Tyne, United Kingdom; c Institute of Agriculture, Faculty of Science, University of Western Australia, Crawley, Western Australia, Australia; d Department of Primary Industries and Regional Development, South Perth, Western Australia, Australia; University of California, Riverside

## Abstract

We describe here the first genome sequence of Arracacha virus Y (ArVY) derived from an arracacha (Arracacia xanthorrhiza) sample originally collected in 1976 in Peru and compare it with other potyvirus genome sequences. It had a 79% nucleotide identity with a 2013 Brazilian Arracacha mottle virus (AMoV) sequence, suggesting that AMoV is ArVY.

## ANNOUNCEMENT

When the samples taken in a 1975 survey of subsistence plantings containing crop mixtures at Umari, Huanuco Department, in the Peruvian Andes were analyzed, two viruses with isometric particles, Arracacha virus A (genus Nepovirus) and Arracacha virus B (AVB; genus Cheravirus) were isolated from arracacha (Peruvian parsnip; Arracacia xanthorrhiza) plants showing pronounced yellow mosaic symptoms in young leaves (1, 2). After a second visit to the same location in 1976, in addition to AVA and AVB being found again in some samples, a third virus with 750-nm flexuous filamentous particles was detected in samples from symptomless arracacha plants. As its particles were typical of the family Potyviridae, which is named after Potato virus Y, the third virus was named Arracacha virus Y (ArVY). A fourth virus with 650-nm filamentous particles typical of the genus Carlavirus was also present. It was first named Arracacha latent virus (3) but was later identified as another Carlavirus, *Potato virus S* (4). In 1981, ArVY was recovered from leaves of arracacha plants that were freeze-dried in 1979 in the United Kingdom. The infected arracacha plants had been propagated serially from tubers since the original tubers from Umari were sent to the United Kingdom for electron microscopy in 1976. The recovered ArVY was mechanically transmissible to several plant species in the families Aizoaceae, Chenopodiaceae, and Solanaceae and was transmitted nonpersistently by the aphid species Aphis gossypii and Myzus persicae (3). ArVY-infected leaf material was freeze-dried in glass vials in 1984 and kept thereafter in what is now called the Fera Science Ltd. plant virus collection in York, England. In 2009, a potyvirus was found infecting symptomatic arracacha in Brazil and called Arracacha mottle virus (AMoV), and a complete genomic sequence of it was obtained in 2013 (5, 6).

In this paper, the methods used were the same as those described previously (7–9). Briefly, an RNeasy kit (Qiagen, UK), was used to extract total RNA from the freeze-dried ArVY-infected leaf material in 2017. Following the manufacturer’s instructions, a ScriptSeq complete plant leaf kit (Illumina, USA) was employed to construct an indexed plant ribosome subtracted sequencing library. Utilizing a 600 cycle V3 kit, this indexed library was sequenced along with others on a MiSeq instrument (Illumina). Sickle (https://github.com/najoshi/sickle) was used in paired-end mode to 3′ trim the resulting 593,960 paired reads to a quality score of 20. The reads were assembled with Trinity v2, with the maximum memory allocation set to 99 gigabytes of RAM, and the process allocated 64 central processing units (10). We used BLAST+ (11) to compare the contigs obtained with the GenBank nonredundant (nr) and nucleotide databases. Next, the extract reads function in MEGAN (12) was used to extract reads of viral origin. One 9,647-nucleotide (nt)-long contig which had been assembled from 439,090 of the paired reads (minimum depth, 10 reads; average depth, 17,918 reads) resembled the genome of a potyvirus. The reads were *de novo* assembled, and the read count was from postassembly mapping back. The ArVY genome had a 79% nucleotide identity to the AMoV genome (GenBank accession number DQ925486). Given that ArVY had a nucleotide identity of >75% with AMoV, both belong to the same potyvirus species (13). As the name ArVY was used first for this virus, it should take precedence over the later name, AMoV.

### Data availability.

The ArVY sequence described here was deposited in GenBank under the accession number MH716807. Raw data were deposited in the SRA under BioSample number SAMN10081144 and SRA run number SRR7873470, which are part of SRA study number SRP162046.
